# Exploring the Multifaceted Therapeutic Potential of Withaferin A and Its Derivatives

**DOI:** 10.3390/biomedicines8120571

**Published:** 2020-12-06

**Authors:** Tapan Behl, Aditi Sharma, Lalit Sharma, Aayush Sehgal, Gokhan Zengin, Roxana Brata, Ovidiu Fratila, Simona Bungau

**Affiliations:** 1 Chitkara College of Pharmacy, Chitkara University, Punjab 140401, India; aayushsehgal00@gmail.com; 2 School of Pharmaceutical Sciences, Shoolini University, Solan, Himachal Pradesh 173229, India; aditisharma31790@gmail.com (A.S.); lalitluckysharma88@gmail.com (L.S.); 3 Department of Biology, Faculty of Science, Selcuk University Campus, Konya 42250, Turkey; biyologzengin@gmail.com; 4 Department of Medical Disciplines, Faculty of Medicine and Pharmacy, University of Oradea, 410073 Oradea, Romania; roxana.gavrila@yahoo.com (R.B.); ovidiufr@yahoo.co.uk (O.F.); 5 Department of Pharmacy, Faculty of Medicine and Pharmacy, University of Oradea, 410028 Oradea, Romania

**Keywords:** Withaferin A, anticancer, autophagy, chaperone

## Abstract

Withaferin A (WA), a manifold studied, C28-steroidal lactone withanolide found in *Withania somnifera*. Given its unique beneficial effects, it has gathered attention in the era of modern science. Cancer, being considered a “hopeless case and the leading cause of death worldwide, and the available conventional therapies have many lacunae in the form of side effects. The poly pharmaceutical natural compound, WA treatment, displayed attenuation of various cancer hallmarks by altering oxidative stress, promoting apoptosis, and autophagy, inhibiting cell proliferation, reducing angiogenesis, and metastasis progression. The cellular proteins associated with antitumor pathways were also discussed. WA structural modifications attack multiple signal transduction pathways and enhance the therapeutic outcomes in various diseases. Moreover, it has shown validated pharmacological effects against multiple neurodegenerative diseases by inhibiting acetylcholesterinases and butyrylcholinesterases enzyme activity, antidiabetic activity by upregulating adiponectin and preventing the phosphorylation of peroxisome proliferator-activated receptors (PPARγ), cardioprotective activity by AMP-activated protein kinase (AMPK) activation and suppressing mitochondrial apoptosis. The current review is an extensive survey of various WA associated disease targets, its pharmacokinetics, synergistic combination, modifications, and biological activities.

## 1. Introduction

Phytotherapy is the frequent therapeutic approach in complementary as well as in traditional medicine since time immemorial. It is utilized by 60% of the global population and has a vital role in the health care system due to ease of availability and reduced cost compared with synthetic compounds. The result of screening showed that many synthetic drugs are derived from natural origin, which is now extensively used in pharmacological therapies [1]. Considering the importance and bringing novel therapies into the market, the herbals have always been at the forefront of synthetic drugs with complex molecular diversity and biological function variation.

Herbals are considered as natural chemical factories for manufacturing natural compounds with structural diversity. The secondary metabolites from plant origin have gained renewed attention because they directly or indirectly interact with multiple cell components, mainly lipids and proteins, thereby altering the functions of dysregulated cells [2]. The evaluation of the phytopharmacological effect of potential herbal drugs is essential for drug discovery and development [3,4,5,6]. Despite the considerable application of natural compounds in drug development, it is assumed that plant-based sources are still unexplored and can be scrutinized for new therapeutic approaches in the modern era. Artemisia annua can reveal the significance of this field (Quinhaosu) derived artemisinin, the antimalarial drug known for its long history in Chinese traditional medicine in the treatment of fevers. The anti-hypertensive agent reserpine is used to remedy snake bite in Indian traditional medicine [7]. Today medicinal plants are evaluated and explored due to modern technologies, including screening and functional assays [8]. There is an escalating demand for novel therapies to treat diseases. There is a growing interest in naturally extracted herbal constituents as targets for potential treatment.

In the Ayurvedic and Unani systems, over the last 3000 years, the genus Withania (family: Solanaceae) has been used indigenously [9]. The distribution is seen widely in the dry regions of the tropical and subtropical areas, extending from the Canary Islands to the Mediterranean region, North Africa, and ending in Southeast/Southwest Asia. Its numerous therapeutic usages have gained modern scientific attention and emerged in World Health Organization (WHO) monographs on preferred medicinal plants. *Withania Somnifera* (Ashwagandha) belongs to the family Solanaceae. It has local names like asgandh, punir (Hindi), Ghodakun, Ghoda (Gujrati), Ashwagandha (Bengali), amukkura, amkulang (Tamil), Pulivendram (Telugu), etc. In Sanskrit, Ashwagandha means “horse’s smell” probably arises from the smell of its root, bear a resemblance to that of a sweaty horse. WS is also known commonly as Indian ginseng and Indian winter cherry. In Latin, *somnifera* is known as “sleep making,” which means sedating properties, but it has adaptogenic properties or sexual vitality strength [3]. Ashwagandha roots are constituted in numerous Ayurvedic, Siddha, and Unani formulations [9].

This medicinal plant’s therapeutic applications include antidiabetic, anti-epileptic, anti-inflammatory, anti-depressant, anti-arthritic, anticoagulant, antipyretic antioxidant, analgesic, regenerating, rejuvenating, and promoting growth [10,11]. The primary chemical constituents include compounds of varying chemical structures viz. flavonoids, withanolides, tannin, and alkaloids. Of these, withanolides have steroidal lactone triterpenoids at C28 position assembled on a reorganized or integral ergostane framework, of which C22 and C26 are in oxidized form resulting in the lactone ring (six-membered) [11].

**Figure 1:**
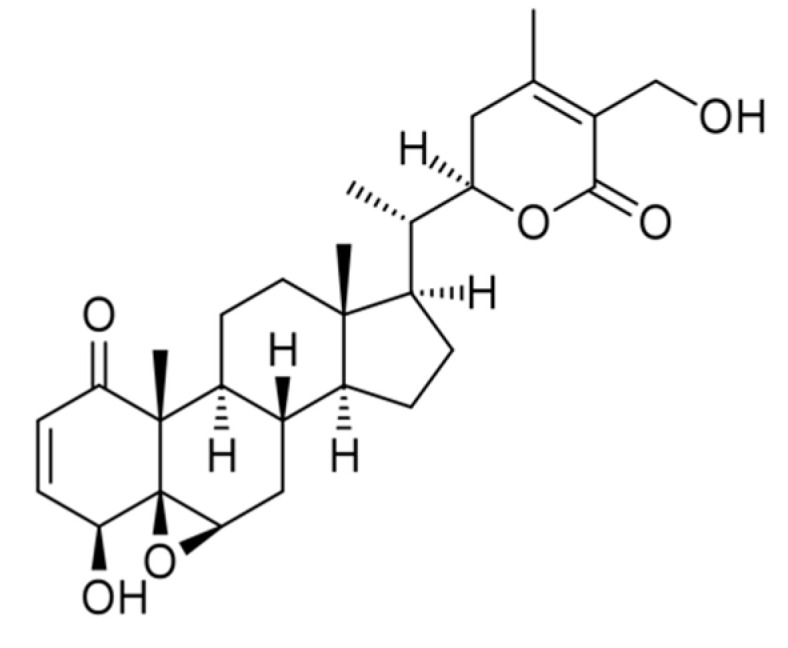
Structure of Withaferin A.

Reverse pharmacology approaches implicated WA as a bioactive compound having the maximum pharmacological potential of Ashwagandha [12] (Figure 1). The analysis of the WA chemical structure displayed 3 positions that may interact with target proteins. Alkylation reactions and nucleophilic site binding reactions occurs through the A-ring at position C3 and the epoxide network in C24 (Figure 1). These sites are most vulnerable for the nucleophilic attack and alkylation reaction; WA interacts covalently with the target proteins [13].

WA evidenced many pharmacological activities, including tumor preventive, antidiabetic, anti-osteoporotic, anti-inflammatory, antiangiogenic effects, cell death inducing, radiosensitizing, and Covid 19 infection. Although the research on molecular mechanisms by which WA attains these pharmacological activities is still going on, various evidence has been indicated, including macromolecules acylation or alkylation or covalent binding to the enzymatic site [13].

The current review is an extensive survey of various WA associated disease targets, summarizing the pharmacokinetics, structural modifications, potential pharmacological activities, and WA formulations, and evaluating also the available evidence to predict the potential targets. More than 150 References, indexed in the most relevant data basis (MDPI, ESEVIER, PubMed, NCBI, Springer, etc.) were found describing parts of our topic.

## 2. Pharmacokinetics and Bioavailability Studies of Withaferin A

Pharmacokinetic (PK) studies provide valuable information on bioactive compounds of herbal drugs. PK analysis is based upon targeted or untargeted metabolites profiling following the oral administration of a single chemical component of the crude drug. The estimation analysis on mice plasma following oral administration of 1000 mg/kg *W. somnifera* root aqueous extract showed 0.4585 mg/kg of WA. The PK data displayed rapid oral absorption of WA with C_max_, T_max_, and T_1/2_ were 16.69 ± 4.02 ng/mL, 20 min, 59.92 ± 15.90 min, respectively. According to one of the studies WA has one and half times more relative bioavailability than other withanolides in *W. somnifera* [14]. The permeability was measured and found that the probability (*P*_eff_) value of WA was 4.05 × 10^−5^, indicating highly impermeable [15]. The oral bioavailability was found to be 32.4 ± 4.8% after 5 mg/kg intravenous and 10 mg/kg oral WA administration. The in vitro analysis indicated that WA could transport across colorectal adenocarcinoma (Caco-2) cells, and it also shows the absence of a P-glycoprotein substrate. The stability studies of WA in gastric fluid, liver microsomes, and intestinal microflora solution showed similar results in male rats and humans with a half-life of 5.6 min.

Moreover, WA reduced quickly, and 27.1% left within 1 h [16]. PK and safety studies of WA advanced stage of cancer were also seen. The phase I study on WA showed that formulation at dose 4800 mg having equivalent to 216 mg of WA, was tolerated well without showing any dose-limiting toxicity. The maximum dose received by cohort patients was four capsules of the WA regimen (TID) [17]. Taken together, the data showed that the administration of WA in advanced stage high grade osteosarcoma patients results in rapid oral absorption and has a good safety profile. Moreover, Phase II clinical trials can be carried at the dose of 216 mg/day [17]. Thus, this natural compound has enormous potential. So, novel targeted drug delivery strategies can be designed to treat various human diseases.

## 3. Structural Modifications of Withaferin A

The pharmacological activity is enhanced by chemical modifications such as hydroxylation or acetylation. Thus, the knowledge of the structure-function association may motivate new drug development [18]. More significant bioactivity, chemoprotective potential, and stability are acquired by alkylated (methyl or ethyl) secondary metabolites [19,20]. Mortalin is a chaperone that inactivates tumor suppressor protein p53 and induces apoptosis deregulation by promoting carcinogenesis. Stimulation of p53 through its complex abrogation with mortalin arrests cancer cell growth in various studies [21,22]. WA interferes with the interaction of mortalin with p53. The docking of 3β-methoxy-Withaferin-A with the binding domain of the mortalin substrate was done. Methylation of WA exerts a crucial influence on its protein binding efficacy, resulting in chemotherapeutic potency attenuation besides developing drug potency [23].

**Figure 2:**
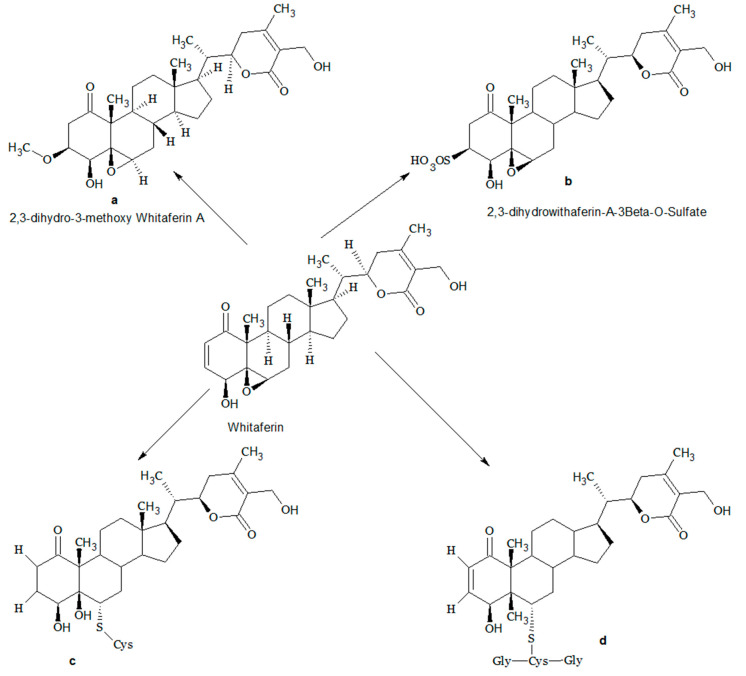
Metabolites of Withaferin A (**a**) 2,3-dihydro-3β-methoxy Withaferin A; (**b**) 2,3-Dihydrowithaferin A-3β-O-sulphate; (**c**,**d**) cysteine and glutathione conjugates of Withaferin A.

Another study showed that two WA conjugates, namely cysteine (CR-591) and glutathione (CR-777) conjugates indicated neuroprotective properties in various neurodegenerative disorders. A nanomolar dose of WA CR-777conjugate reversed mesencephalic neuron injury caused by (1-methyl-4-phenylpyridinium (MPP+), alpha-synuclein (α-Syn), 6-hydroxydopamine (6-OHDA). Moreover, WA CR-777 conjugate maintains neurite integrity, and the overexpression of α-Syn caused by 6-OHDA was reduced. These compounds activate the PI3K/mTOR pathway, which downregulates the oxidative stress, suppresses the TAU phosphorylation, caspase three expression, and aggregation of α-Syn exhibiting neuroprotective properties [24]. An analogue of WA, 2, 3-dihydro-3β-methoxy (3βmWi-A) having β-methoxy group substitution showed no cell cytotoxicity and at higher concentrations well tolerated. It has a protective action in normal cells against ultra-violet (UV), oxidative and chemical stresses and through pro-survival signaling [25]. Another analogue, 2,3-dihydrowithaferin A-3β-O-sulphate, showed a 35-fold increase in vitro cytotoxicity compared to WA against various human cancer cell lines [26]. The different analogues of WA are shown in Figure 2.

## 4. Pharmacological Activities of Withaferin A

### 4.1. Anti-Cancer Activity

The anticancer activity on WA was commenced around the 1970s [27]. Since then, WA’s anticancer activity was demonstrated in many cancer cells such as multiple myeloma, neuroblastoma, leukemia, glioblastoma, ovarian, breast, colon head, and neck cancer [12]. The various molecular mechanisms involved target cytoskeleton structure and proteasomal pathway by altering oxidative stress, promoting apoptosis, and autophagy, inhibiting cell proliferation, reducing angiogenesis and metastasis progression. It regulates heat shock proteins, nuclear factor kappa B (NF-κB), and other oncogenic events [28]. Here, we discuss the chemo-preventive effects of WA on multiple organs.

#### 4.1.1. Breast Cancer

Breast cancer is a severe malignancy affecting thousands of women globally. More than 40,000 women in the United States alone are expected to have breast cancer in 2020 [29]. The onset of breast cancer depends on the sex hormone estrogen, participating in tumor growth (by its receptor nuclear estrogen receptor). The two types of estrogen receptor (ER) genes are involved in tumor formation, namely ERα and ERβ. In breast cancer, the critical role is played by ERα. Thereby it is targeted by many pharmacological therapies. Endocrine treatment may reduce the tumor progression by decreasing the endogenous estrogen levels or interfere with ERα stimulation (e.g., by inhibiting enzyme aromatase). This results in tumor disappearance [30,31]. A study on mice with breast cancer revealed WA cytoplasmic action through compacting DNA molecule and splitting the enzyme poly-(ADP-ribose)-polymerase [32]. Another study has demonstrated that WA regulates the signal transducer pathway and activates transcription 3, attenuates IL-6 in inducible (MCF-7 and MDA-MB-231), and constitutive (MDA-MB-231) cell lines. In MDA-MB-231and MCF-7 cells exposed, WA displayed downregulation of STAT3 transcriptional activity with/without stimulation of interleukin 6 (IL-6) in both cells. The apoptosis was also triggered by WA and can impede cell migration by regulating STAT 3, thus showing therapeutic effect [33]. Another study showed that WA when investigated in mitochondrial dysfunction associated with reactive oxygen species (ROS) generation, resulted in apoptosis of cells. The WA treatment decreases the oxidative phosphorylation as well as also suppresses the activity of complex III. On treatment with WA, DNA impaired variant mitochondrial Rho 0 cell line and 40 embryonic fibroblast-derived from Bax/Bak knockdown cells displayed more resistance than wild-type cells [34]. WA suppresses human breast cells’ proliferation by decreasing the proliferating cell nuclear antigen (PCNA) expression [35]. WA enhances the vimentin phosphorylation at serine-56 residue, thereby inhibiting the proliferation in 4T mouse mammary tumor cells [36]. WA in DNA double-strand break (DSB) inhibits the single-strand annealing sub-pathway (SSA) through heat shock protein (HSP90) downregulation [37]. To block autophagy flux, WA inhibits lysosomal activity and induces apoptosis of breast cancer cells [38]. WA action leads to the aggregation of autophagosomes (protein expressions associated with autophagy). The inadequate fuel recycling and tricarboxylic acid substrate results due to the autophagic flux inhibition inducing phosphorylation impairment. WA treatment decreases the lactate dehydrogenase (LDH) expression, increases AMP protein kinase activation, and reduces adenosine triphosphate [39].

#### 4.1.2. Ovarian Cancer

In human ovarian cancer cell lines (SKOV3 andCaOV3), WA arrest the G2/M phase cell cycle [40]. It downregulated the Notch-3/Akt/Bcl-2 signaling mediated cell survival, thereby causing caspase-3 stimulation, which induces apoptosis. Withaferin-A, combined with doxorubicin, and cisplatin at suboptimal dose generates ROS and causes cell death [41,42,43]. In another study using the A2780 cell line, Xenografting resulted in mortality decreased by WA. It reduces the cytosolic and nuclear levels of NF-κB-related phospho-p65 cytokines in xenografted tumors [44]. Another study showed that ovarian cancer xenografting induced cardiac cachexia, causing loss of the heart’s normal functioning, systolic, and diastolic dysfunction. WA treatment improved heart weight and preserved systolic function, but the partial improvement was seen in diastolic dysfunction. Tumor cells induce AT_1_R pathway mediated pro-inflammatory markers and formation of MHCβ isoform, which was ameliorated by WA [45]. In nude mice, securin overexpression leads to cellular transformation and tumor development. Knockout securin mice show no tumor development and reverse the cancer phenotype. WA alone or with Cisplatin decreases the expression of securin and showing antitumor effects [46].

#### 4.1.3. Prostate Cancer

Initially, the pathogenesis linked with prostate tumors is an androgen; however, numerous patients also progresses to androgen-independent (metastatic castration-resistant) [47]. The stem-like characteristics are shown by androgen-independent PCa cells (mCRPC) DU-145 and PC-3), whereas exhibited by androgen-dependent PCa cells (e.g., LNCaP) [48,49,50]. The side populating cells of xenograft tissues and human PCa cell lines exhibited more epithelial-mesenchymal transition (EMT) and comparably more violent than homologous bulk population cells [51]. Thereby, EMT is very closely linked with the mCRPC formation. In prostate tumor cells, WA binds vimentin and induces cell death, but no cell death was seen in normal fibroblasts. It raises the level of c-Fos and ROS generation and decreases the FLIP level, probably resulting in cytoskeletal architecture degradation. Thus, WA can be used as a pharmaceutical agent that effectively kills cancer stem cells (CSCs). These CSCs are different from other cancer cells, as their presence within the tumor mass mediates chemoresistance by regenerating tumors [52]. WA interacts directly with vimentin by causing an alteration in cysteine residue (Cys328), forming an aggregation of vimentin filaments and together with F-actin, causing disruption of vimentin cytoskeleton [53,54]. This is followed by alteration in cell shape, reduced motility, and upregulated phosphorylation of vimentin at Ser38 [55].

The observations evidenced that WA can efficiently target metastatic tumor cells [36]. In cell lines of pancreatic cancer, Panc-1, BxPc3, and MiaPaCa2, WA inhibit Hsp90 chaperone activity, disrupting Hsp90 client proteins, thus showing antiproliferative effects [56]. Another study has shown the efficacy of WA against the CaP iPten-KO model. The role of the metastatic process Akt pathway, Pten deletion, mutation, EMT was seen commonly in metastatic prostate tumors. WA abrogated HG-PIN formation and ameliorated the progression of the Pten-deficient tumor to adenocarcinoma. WA inhibited PI3K/AKT pathway. The AKT-mediated Par-4 and FOXO3A proapoptotic proteins were increased in Pten-KO mice supplemented with WA. Immunohistochemical analysis displayed decreased pAKT expression and the β-catenin and N-cadherin epithelial-to-mesenchymal transition markers in WA-treated tumors control [57]. DNA damage response is initiated by Telomere shortening, which results in senescence and apoptosis [58,59]. The cancer cells run away from the shortening of telomere by enzyme telomerase [60,61]. In ALT cells, WA caused severe damage to telomere by downregulation of shelterin proteins (TRF2 and POT1). It was observed that WA caused cytotoxicity to ALT cells with no effect on telomere and telomerase activity length. By telomerase-independent mechanisms, it kills TEP cells, as reported previously [62]. Another study showed that WA significantly inhibited pAKT expression and facilitated FOXO3a/Par-4 mediated tumor inhibition in TRAMP mice [63]. One of the critical sources of Cancer is mutations in the Wnt pathway that control cancer hallmarks like metastasis and immune evasion [64,65,66,67]. Wnt signaling pathway hyperactivation triggers the transformation of normal cells to malignant [68,69].

Interestingly, WA analog 3-azido WA can suppress the Wnt pathway indirectly and thus prevent the transformation of Cancer [70]. WA and its metabolite 3-azido-WA also increase protein, namely proapoptotic prostate cancer response-4 (PAR-4) in androgen-refractory prostate cancer cells [71]. It modulates the β-catenin phosphorylation in the Wnt pathway. In particular, an increased amount of PAR-4 downregulates the Akt kinase activity induces the glycogen synthase kinase 3 beta (GSKβ) activation. This phosphorylates βcatenin is known to obstruct the Wnt signaling pathway [70]. Another study showed WA intraperitoneal administration (0.1 mg) resulted in significant suppression of circulatory free fatty acid and fatty acid synthase expression, ATP citrate lyase, and carnitine palmitoyl transferase 1A proteins in vivo prostate studies [72].

#### 4.1.4. Colorectal Cancer

Another common cancer is colorectal cancer (CRC), the fourth leading cause of mortality worldwide [73]. Despite the incredible progress of efficient chemotherapeutic agents, the drug resistance occurred, determining the non-success of chemotherapy, with escalated toxicity to the gastrointestinal tract, skin, and bone marrow [74,75]. In human colorectal cancer cells, WA generates ROS followed by the activation of Nrf2, HO-1, NQO1 pathways, and upregulating the expression of the c-Jun-N-terminal kinase (JNK), the upstream regulator of Nrf2. This resulted in cell death induction by blocking the disruption of tumor suppressor geneTAp73. WA induces in G2/M phase cell cycle arrest in HCT116 and SW480 cells of colorectal cancer [76]. It delayed mitosis by interfering with the proteasomal disruption of Mad2 and Cdc20, necessary constituents of the spindle complex [76].

Moreover, it decreased expression and proliferation of Notch-1 in human colon cancer cells [77]. In colorectal cancer, EMT causes AKT and Notch1 activation. As EMT leads to this type of cancer, therapies that target AKT/Notch1 pathways and prevent metastasis are at the top of the current research paradigm. The WA effect was studied on colitis regulated colon mouse model and other mouse models of spontaneous intestinal carcinogenesis. WA effectively inhibits the development of intestinal polyp and colon carcinogenesis; it showed also the downregulation of pro-survival signaling markers (NFκB, Notch1, and pAKT) and reduced the proliferative markers [78]. The chemoprotective action on spontaneous and inflammatory colon carcinogenesis patterns was performed on transgenic mice models; WA treatment demonstrated declined tissue inflammation and adenomas. In the molecular analysis, WA down-regulates the expression of Notch1, pAKT, and NF-κB and other markers of inflammation (interleukin-6 (IL-6), tumor necrosis factor alpha (TNFα), and cyclooxygenase-2) [79], highlighting that this therapeutic agent has a vital role in colon carcinogenesis prevention. Thus, it can be further explored for clinical utility.

#### 4.1.5. Lung Cancer

In lung cancer cells (A549 and H1299), WA pre-treatment showed suppressed cell adhesion, migration, and invasion. Using immunofluorescence, qRT-PCR, and western blot analysis, it was proved that WA downregulated EMT induced by tumor growth factor beta 1 (TGFβ1) and TNFα expression in both cells. WA also suppresses Smad2/3 and NF-κB phosphorylation and nuclear translocation [80]. In another study on A549 cells, WA causes dose-dependent apoptosis. JC-1 staining of cells treated with WA showed declined MMP and was accompanied by caspase-9 and caspase-3 activation, the apoptosis leading players [81]. WA arrested the G0/G1 phase of lung cancer (A549) cells, which further suppresses phosphatidyl inositol-3 kinase (PI3K)/Akt pathway and decrease the Bcl-2expression. WA also displayed a dose-dependent decrease of metastatic lung nodules. WA is an effective anti-lung cancer agent as well as also controls the growth of CSC. It also inhibits the spheroid formation in lung cancer by suppressing the mTOR/STAT3 pathway [81].

### 4.2. WA-Responsive Proteins in Cancer

Carcinogenesis is a process with multiple stages that involve the deregulation of various physiological and biochemical cascades that control cells’ growth and survival and their apoptosis. In this context targeting various signaling mediators that lead to tumor growth is advantageous to be discussed. The electrophilic nature and its interaction with the nucleophilic group results in inducing electrophilic stress, thereby it shows WA as a Michael addition electron acceptor. These free radicals will cause damage to the mitochondria leading to its apoptosis. There is an increase in the exogenous ROS levels dramatically during drug uptake or environmental stress, such as UV radiation [82]. WA treatment forms ROS in various models of Cancer. WA interaction to Keap1 causes increased NRF2 protein levels, which mediate the antioxidant protein expression protecting the cell against the effect of oxidative damage [83].

Meanwhile, various proteins are targeted by WA from the anti-stress pathway and increasing the ROS level. This augmented ROS level further activates antioxidant pathways and causing ROS/cyto-protection imbalance. This finally decides the fate of cancer cells. WA treatment enhances four oxidative stress response proteins that decrease the oxidative damage following WA treatment and re-establish homeostasis. The upregulated proteins in oxidative stress response upon WA treatment include heme oxygenase, one iron-sulfur, aldose reductase, and sepiapterin reductase. Various proteins are downregulated glutathione peroxidase 1, phospholipid hydroperoxide [84,85].

Following oxidative stress and activation of the ubiquitin-proteasome system (UPS) is activated by the Nrf2 transcription factor to remove oxidized proteins and restore homeostasis. Targeting UPS induces more proteotoxic stress in cancer cells. WA treatment upregulates five proteins related to ubiquitin-proteasome includes beta type-1 (PSB1 human), Proteasome subunit alpha type-2 (PSA2 human), 26S proteasome regulatory subunit 10B, Ubiquitin carboxyl-terminal hydrolase (UBP24 human), and Proteasome activator complex subunit-4 (PSME4 human). The WA-target proteins that degrade include Proteasome subunit beta type-10 and type-5, AAA+ chaperone p97, USP24 human isozyme L5 [86,87].

UPS is responsible for cellular removal of proteins, although cells also have an extra protein removal system known as autophagy that disrupts cellular components and the deposited aggregates of protein during UPS attenuation [88]. Autophagy is an adaptive cellular stress response. The treatment with WA disrupts and block autophagy functions [38]. Therefore, autophagy markers that upregulate during WA treatment include mitochondrial import receptor subunit TOM22 homolog, SNARE-associated protein Snapin, ras-related protein Rab-24, tubulin beta chain, histone deacetylase 6, Annexin A4, Tubulin Beta [89,90].

Another transcription factor known as heat shock factor-1 (HSF-1) regulates protein folding and repair [91]. This factor induces stress and also binds with DNA and causes the stimulation of heat shock response elements. WA has a potential antitumor activity, indicating very powerful ER-stress causing properties, assumed from HSF1 reporter assay [92]. HSF1 is marked as a promising WA binding target, further leading to the suppression of genes controlled by HSF-1, such as FKBP4 HSP13 and DJC10. The proteins exaggerated treatment with WA includes chaperone, ER-associated degradation, and protein folding enzymes (isomerase and reductase). Proteins associated with chaperone related protein unc-45 homolog A, heat shock 70 kDa protein 1A; 1B; 13, DnaJ homolog subfamily A member 2, BAG family molecular chaperone regulator 2. The protein targeted by WA and linked with the enzymatic activity of isomerase and reductase include Peptidyl-prolyl cis-trans isomerase, Peptidyl-prolyl isomerase domain, NIMA-interacting 1, and WD repeat-containing protein 1. Proteins that target ER-associated degradation include alpha-mannosidase protein 3, Homocysteine inducible ER, AAA+ chaperone p97, transport and golgi organization protein 1 homolog [84]

At the time of weak cellular stress response initiated by WA, Protein translation must be restrained to avoid new misfolded proteins’ aggregation. WA has a role in protein translation machinery, which increases eukaryotic translation initiation factor (IF) 2A, which inhibits cancer cells’ proliferation. However, subsequent interaction of WA with IF5A1 (the eIF5A regulon) and IF4B triggers cell death [93].

Moreover, WA also targets the cytoskeleton properties, which include Vimentin, annexin A2, and β-tubulin. Vimentin (VIM) plays the leading role in holding and anchoring organelles by making connections of the nucleus, mitochondria, and endoplasmic reticulum in the cytosol. Various kinases have shown binding with vimentin such as ROKa, Raf-1, phosphorylated ERKs, PKCe [94,95,96]. Alternatively, vimentin interacts with 14-3-3 proteins that might obstruct signaling cascades that mediate cell cycle progression, signal transduction, and apoptosis; it also binds with HSP90. Furthermore, there is an enhanced vimentin expression in cancerous cells, and it is correlated with EMT, metastasis with poor prognosis thereby resulting in reduced patient survival [54,97]. When binds with vimentin, it inhibits the assembly and intermediate filament network and leads to eventual viability [98]. Annexin A2 (ANXA2) is a calcium-dependent protein, supporting endocytosis, adhesion, and metastasis. It is present abundantly in a broad range of cancers. WA, when binds to annexin A2 core domain, causes suppression of actin polymerization and subsequent limit the migration of the cancer cells [99,100]. Literature also reports that WA arrests the G2/M and G1/S transition cell cycle phase [32].

The targets identified are Dual specificity protein kinase TTK, Nucleoporin Nup43, Protein phosphatase 1B SRRT human, WAPL human, NIPA human, MCMBP human [84]. WA also disrupts of NFκB signaling pathways. It is an important pathway that is dysregulated in various types of Cancer [101]. This suppresses the gene transcription of many downstream genes involved in inflammation includes MCP1, Interleukin-1 (IL6), and IL8, etc. The proteins participated in NFκB signaling and downregulated by WA include nuclear factor NFκB p105 subunit, Transcription factor p65, signal co-integrator one complex subunit 2, Coiled-coil domain-containing protein 22, COMD3 human. NF-kB participates in the activation of proteins involved in cell growth, cell survival, angiogenesis, and decrease vulnerability to apoptosis [84,102]. NF-kB driven proteins expression stimulation is controlled tightly. Under quiescent conditions, it is an inactive form in the cytoplasm with its inhibitor IkB, masking the nuclear localization process. Inflammatory mediators, and microbial pathogens trigger specific cognate receptors and stimulate NF-kB. This event induces the stimulation of the IkB-kinase (IKK) complex leading to its proteasomal degradation [102]. WA inhibited NF-kB signaling, which includes chronic lymphocytic leukemia (CLL), acute myeloid leukemia (AML), and multiple myeloma (MM) [13].

**Figure 3:**
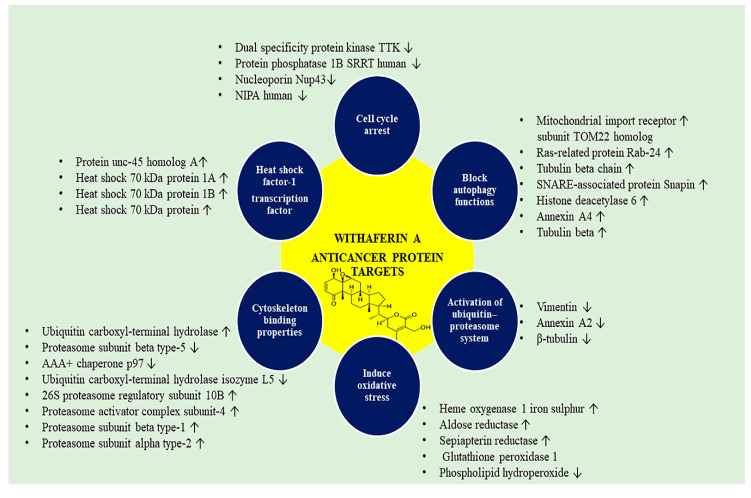
Withaferin A associated anticancer protein targets.

Cancer stem cells (CSC) are the self-renewal and tumor-initiating cells. The rising trend in the failure of chemotherapeutic drugs and the reappearance of cancer has contributed to cancer stem cells’ growth in growing tumors. The main priority is the eradication of these cancer stem cells in modern endeavors of anticancer drug discovery. In the cells of pancreatic ductal adenocarcinoma nestin overexpression, a stem cell marker rises cell motility and results in phenotypic changes, whereas endogenous nestin knockdown decreases cell migration well as cells also retain its epithelial phenotype. WA possesses anti-nestin activity, which suggests its potential in targeting pancreatic CSC [103]. Another study reported [104] the suppression of mammosphere activity, the aldehyde dehydrogenase 1 (ALDH1) activity, elevated CD44, and reduced CD24 levels in breast cancer cells (MCF-7 and SUM159). This poly-pharmaceutical response rationalizes its anticancer mechanism. Chemo proteomic approach shows WA binds and interacts with numerous proteins targeting various protein signaling cascades of cancer. The various anticancer targets are shown in Figure 3.

#### Oxidative Stress Response and Red Cell Proteins

WA was reported to induce ROS production causing oral and colon cancer cells apoptosis [105,106]. In cancer cells, WA induces G2/M cell cycle arrest, antiproliferation, apoptosis, mitochondrial membrane depolarization, caspase activation, migration inhibition and phosphorylated histone H2A.X based damage to DNA [107]. WA inhibits the activities of MMP-2, MMP-9, causing antioxidant gene expression and activation of MAPK. In oral cancer cells N N-acetylcysteine (NAC) pre-treatment suppressed the inhibitory migration alteration and various pathways activated following the WA treatment proving that ROS plays a crucial role in inhibitory migration induced by WA [108].

Moreover, in tumor cells, the cell surface glycoproteins and glycolipids have changed composition of carbohydrates causing aberrant cell-cell recognition, antigenicity, cell adhesion, and malignant cells invasiveness [109]. Glycosyl residues present on the cell surface controls epithelial growth and cell proliferation [110]. During carcinogenesis, the levels of plasma glycocongugates are elevated at the expense of erythrocyte membranes with the depletion in epithelial cell surface carbohydrates [109]. The loss of erythrocyte membrane glycoproteins was due to increased degradation or due to increased shedding into circulation or reduced synthesis. Neoplastic transformation causes increased levels of plasma sialic acid through the secretion or shedding from the tumor cell [111,112]. As the tumor mass grows, it may contribute to elevated fucose concentrations as a result of glycoproteins release or due to host treatment causing cell damage. WA treatment protected the red blood cell membrane integrity by maintaining glycocongugates level during carcinogenesis. Oxidative stress alters the membrane bound enzymes, which is important for cell lysis [113]. It has been showed that any changes in red cell fragility induces change in Na^+^/K^+^-ATPase activity [114]. Tumor bearing hamster’s red blood cells were more fragile when compared with control hamsters. This increased fragility may be due to their lipid content alteration and increased oxidative stress. WA significantly restored membrane TBARS levels, Na^+^/K^+^-ATPase activity and osmotic fragility in red cell [115]. Human umbilical vein endothelial cells (HUVECs) as well as endothelial cell line (EA.hy926) when treated with WA showed increased heme oxygenase (HO-1) expression, an antioxidant gene. This gene is transcriptionally regulated by NF-E2-related factor 2 (Nrf2) transcription factor which senses chemical alteration in the cell and regulates transcriptional responses thus maintaining chemical homeostasis via antioxidant gene expression. WA upregulates and increase the nuclear translocation of Nrf2, inducing HO-1 expression in endothelial cells thereby re-establish cell homeostasis [116].

### 4.3. Anti Diabetic Activity

Diabetes is a metabolic, endocrine disease where the glucose supply and utilization are mismatched. The pathogenesis includes elevated oxidative stress, which disrupts the pancreas histology and causes beta-cells destruction. Insulin-dependent or type I diabetes mellitus depends totally on insulin replacement therapy as the therapeutic strategy. There are various parts and parcel complications of diabetes, such as obesity, organ sepsis, and microvascular and macro-vascular diabetic complications [117,118]. Another evidence reported that WA leads to potential alteration in glucose metabolism and lipid profiles. In diabetic mice, it suppresses inflammation and stimulates weight loss, contributing to elevated insulin sensitivity [119]. WA attenuated mouse hepatic steatosis, although antidiabetic drug rosiglitazone has a beneficial effect on insulin sensitivity but does not show improved liver and weight loss effects. Inflammatory mediators (TNF-α, IL6, and resistin) play a significant role in obesity. They promote insulin receptor substrates 1 (IRS-1) phosphorylation that negatively controls the signaling of insulin. They were significantly decreased by WA treatment. Previous literature has shown the downregulation of insulin signaling gene expression (insr, pi3k irs1, slc2a4, and irs2) in diabetes. WA treatment upregulated the mRNA expression of insr, insr, pi3k, and irs1 while treatment with rosiglitazone increased insr, irs1, and slc2a4 expression. WA insulin-sensitizing potential seems to have occurred due to its anti-inflammatory action that indirectly affects insulin signaling events, upregulating adiponectin, preventing the phosphorylation of PPARγ [119]. The studies reported attenuation of streptozotocin-induced type 1 diabetes by WA. Oxidative stress stimulates the NF-κB axis, following inflammatory mediators’ production that induces pancreatic islet cell destruction. STZ induces alkylation of DNA that ultimately leads to the β-cell dysfunction. Apoptotic morphological changes occur by Caspase 3 upregulation, including DNA fragmentation, membrane blebbing, apoptotic body formation, and cytoplasmic and nuclear condensation [120]. This results in the disruption of pancreatic insulin-secreting β-cells, resulting in hyperglycemia [121,122]. WA intervention abrogates nitrosative stress by lowering tissue nitrile levels. WA was considered as a potential molecule to ameliorate T1DM by caspase three expression, reduction in fragmented DNA, decrease in the concentration of TNF-α, and IL-6 [122]. In acute liver injury induced by acetaminophen, WA activates the Keap1/Nrf2 pathway and mediates the hepatoprotective effect. The Nrf-2/NFκB signaling imbalance leads to a cascade of oxidative stress causing liver damage [123]. Moreover, in another study, also WA proved to be anti-inflammatory and antioxidant. It restores the impaired insulin resistance and corrects the endothelial function [124]. Taken together, these results implicated that TNF-α, IL6, and Nrf-2/NFκB are an essential target of WA.

### 4.4. Neuroprotective Activity

In the central nervous system (CNS), Aβ accumulation contributes to neurodegeneration. WA 0.5–2 μM decreases the amyloid beta (Aβ) aggregation induced by Tat and cocaine with no cytotoxicity in the cell cultures. WA treatment decreases cytoplasmic vacuoles and dendritic beading. Moreover, this Aβ accumulation in an HIV patient’s brain contributes to cascades of neurological disorders that drive aging or related dementias [125]. Moreover, WA is reported to block the acetylcholesterinases and butyrylcholinesterases enzyme activity in in vitro assay.

The hydrolytic activity acetylcholinesterase disrupts neurotransmitter acetylcholine, thereby forming choline and acetate. The role of butyrylcholinesterase still to be explored. As acetylcholine has a significant role in cognitive diseases, acetylcholine’s upregulation ameliorates the cognitive deficits in Alzheimer’s disease (AD) [126]. The neuroprotective ability of WA (50 mg/kg b.w) demonstrated a resurge of dopamine (DA) and homo vanillic acid (HVA) in substantia nigra and striatum. The reduced level of these catecholamines leads to motor deficits. The increased level of DA and HVA suggest the neuroprotective potential of WA [127]. Traumatic brain injury (TBI) has increased morbidity and mortality rates worldwide, making it a significant public health concern. WA significantly enhanced neurobehavioral function and reduced histological alteration in tissues after injury; it reduces the disruption in the blood-brain barrier and edema in the brain via decreasing apoptosis in endothelial cells. WA attenuate the levels of neuroinflammatory mediators (TNF-α, IL-1β, and IL-6). This regulation regulating microglial activation can be used as a therapeutic regimen for recovery after traumatic brain injury [128]. 

In AD, activation of microglia is achieved by interaction with Aβ oligomers and Aβ fibrils, which causes an inflammatory reaction by stimulating NLRP3 and nuclear factor NF-κB pathway inducing the release of pro-inflammatory cytokines and chemokines [129,130]. By phagocytosis, Aβ fibrils are engulfed by microglia, and these fibrils are degraded by neprilysin and insulin-degrading enzyme. In patients of AD, stimulation of NLRP3 and NF-κB pathway block the Aβ phagocytosis causing increased Aβ fibrils accumulation, thereby forming a self-perpetuating loop, resulting in neuroinflammation [130]. NF-κB expression was inhibited with WA’s treatment, which plays a vital role in the cascade of inflammatory cytokines. The downregulation of JAK and STAT and upregulation of IKBKB and IKBKG was seen [131].

Leucine-rich repeat kinase 2 is a massive protein mutated in neurological patients with Alzheimer’s disease and Parkinson’s disease (PD). This protein is stabilized by chaperone HSF90 and also with co-chaperone Cdc37. When treated with WA, the N9 microglial cell line decreases the LRRK2 and disrupts the HSP90, Cdc37, which results in destabilization and reduced concentration of LRRK2 [86]. A more beneficial anti-inflammatory role of WA was seen in transgenic mice with TAR–DNA binding protein (TDP43). These mice have a neurodegenerative disease similar to amyotrophic lateral sclerosis and indicate activated microglia with neurotoxic and pro-inflammatory phenotypes. TDP43 expression, NF-kB subunit p65 expression, was found to be increased in the spinal cord. WA treatment improved motor neuron deficits and decreased NF-kB dependent inflammation and thereby decreasing the disease phenotype. WA (10 to 40 mg/kg ip) also displayed anxiolytic efficacy, as measured by increased exploratory time in the open arm in the elevated plus-maze [132]. Altogether this data confirms that WA has enormous potential as a natural neurotherapeutic agent in ameliorating cognitive deficits associated with AD, PD, ALS; thus, its application in other models of neurodegenerative diseases deserves to be investigated.

### 4.5. Cardioprotective Activity

Myocardial infarction (MI) is a significant health problem globally and the leading cause of death. WA 1mg/kg decreased the apoptotic cell death, upregulation of protein Bcl-2, and thereby stimulating the mitochondrial antiapoptotic pathway. The in vivo study evidenced WA low dose 1 mg/kg, in mice has a beneficial effect against MI injury, but a higher dose of 5 mg/kg, in mice was not at all protective and deteriorated cardiac cells. WA may affect the Bcl-2/Bax ratio, induced AMPK activation suppressing mitochondrial apoptosis, and thus showed protective cardiac function. Thus, WA has therapeutic usage in patients having cancer enduring cardiovascular system disorders [133].

Fibroproliferative disorders are a type of tissue injury linked with stimulation of collagen synthesizing cells, inducing type I collagen synthesis and deposition [134]. This result due to collagen I gene expression transcription and the escalated collagen I mRNAs half-life [135]. WA disrupts the vimentin filament network in endothelial cells and astrocytes. Vimentin filaments interact and stabilize the type I collagen. WA participates in the regulation of transcriptional and posttranscriptional type I collagen and inhibits the stimulation of TGF, block activation of NF-kB.

Myocardial fibrosis is linked with the accumulation of collagen fibers in the cardiac interstitium, seen in various cardiac disorders: myocardial infarction, hypertensive heart disease, idiopathic interstitial cardiac fibrosis, hypertrophic cardiomyopathy, and decompensated congestive heart failure. The fibrosis disrupts cardiac function resulting in heart failure [136,137,138,139]. The studies also showed the antiplatelet, profibrinolytic, and anticoagulant WA (0.09 ug to 4.71 ug/mouse). In TNF-α stimulated human umbilical vein endothelial cells, WA’s effect was evidenced on the plasminogen activator inhibitor type 1 (PAI-1/t) expressions and tissue-type plasminogen activator (t-PA). WA blocked thrombin-catalyzed fibrin polymerization and also the platelet aggregation induced by ferric chloride. WA increased bleeding time and also suppressed TNF-α induced PAI-1 synthesis in vivo and ex vivo studies. Moreover, the PAI-1/t-PA ratio was reduced by WA [140]. Altogether, these results evidenced that WA has cardioprotective potential; considering its safety and efficacy, there is a need for clinical trials to support its therapeutic role in heart diseases.

### 4.6. COVID 19

The proper treatment of cancer patients with the potentially compromised immune system and SARS-CoV2 infection is a serious issue faced by oncologists [141]. Data from four hot spots regions, namely China, United States, Italy, and Spain, showed that patients are admitted to the intensive care unit (ICU) and need mechanical ventilation for life support [142,143,144,145]. Covid 19 infection is associated with life-threatening immune reaction, and there is a release of pro-inflammatory cytokines termed as cytokine storm [146]. In a metastatic ovarian cancer model, WA decreases the pro-inflammatory cytokines (IL-6, TNFα, IL-8, IL-18) [44]. The realm of possibility showed that WA reduces the cytokine storm intensity due to its anti-inflammatory properties reported. Two research groups demonstrated that withanolides (such as WA) can bind with the S protein receptor-binding domain of virus, thereby inhibiting the viral binding with the host’s ACE2 receptor [147,148]. Another group indicated that WA and withanone bind with coronavirus’s main protease, but WA has low a binding affinity compared to an established inhibitor of N3 protease in docking scores [149]. WA treatment decreases the angiotensin II receptor type 1 mRNA expression compared to control groups. Based on these findings and previously reported studies, WA treatment was observed to alter lungs’ ACE2 expression under tumor-free and bearing states. No significant results in lungs ACE2 mRNA expression were there. TMPRSS2 is involved in S protein priming, which causes the cleavage of S protein and thereby allowing viral and cellular membranes fusion. SARS-COVID enters via interacting with angiotensin-converting enzyme 2 (ACE2) WA bind at the catalytic site of TMPRSS2 and able to alter its allosteric site. Therefore, WA can be a potential therapeutic compound to prevent the COVID-19 spread by blocking ACE 2 expression and reducing pro-inflammatory cytokines [150].

### 4.7. Anti Hepatitis Activity

Nonalcoholic steatohepatitis (NASH) is known as the advanced form of nonalcoholic fatty liver disorders that collectively results in the risk of liver cirrhosis and carcinoma [151,152]. Over intake of fatty acids leads to the formation of toxic lipids inducing inflammation, ER stress, hepatic oxidative stress, and hepatic cell death [153]. Out of all the lipids, ceramides get accumulated in the blood and tissues. WA decreases oxidative stress, displayed by its nuclear factor erythroid related factor 2 pathway and heme oxygenase (HO-1) expression, thereby also ameliorating acetaminophen-induced liver injury. WA 5 mg/kg improved pathologies associated with NASH, such as hepatic steatosis, fibrosis, and inflammation. Various pathways involve kelch like ECH associated protein 1, glycogen synthase kinase 3 [154]. These findings suggest that WA effectively protects cells from NASH, although further studies are still required to know the exact mechanism. This could help in the repurposing of this WA with its potential role in treating NASH.

### 4.8. Osteoporosis

Osteoporosis is a skeletal bone disorder characterized by an imbalance in bone resorption and formation [155]. WA (5 and 10 mg/kg) upregulates the osteoblast-specific transcription factor expression, promoting osteoblast proliferation and differentiation. WA down-regulates the inflammatory cytokines. In osteoclast, also known as bone-resorbing cells, WA suppresses osteoclast number by downregulating the expression of tartrate-resistant acid phosphatase (TRAP) and receptor activator of nuclear factor kappa-B ligand (RANKL) and osteoprotegerin (OPG). Furthermore, WA also inhibits NF-kB signaling, activated nuclear p65-subunit of NF-kB, and stabilizes RunX2. Thus, promoting the activity of osteoblastic bone-forming cells [156]. These findings indicated that WA prevents osteoporosis by downregulating TRAP and RANKL, thereby inhibiting osteoclast differentiation.

## 5. Formulations Prepared from Withaferin A

Dexamethasone and WA gold nanoparticles were able to inhibit the epithelial-mesenchymal transition in tumor cells, preventing metastasis by inhibiting mouse melanoma tumors, thereby reducing mice’s mortality rate Glucocorticoids receptor-dependent selective cytotoxicity occurs using this metallic nanoparticle formulation [157]. In one study, mannosylated liposomes (ML) were used for encapsulation of WA (adjuvant-induced) for targeting synovial macrophages in a rat model of arthritis. With the help of confocal microscopy, ML-WA showed robust internalization of synovial macrophages. Moreover, osteoprotegerin production was upregulated after the treatment, and there was no degradation of cartilage and bone erosion. The study suggested, ML-WA has enormous potential for reducing bone resorption and inflammation [158].

A new liposomal efficient drug delivery system was developed to target angiogenic endothelial cells and CD13 positive cancer epithelial cells using homing peptide (NGR). NGRKC16-lipopeptide liposomes are encapsulated with WA, which leads to the apoptosis of CD13-positive pancreatic cancer cells and angiogenic endothelial cells. Therefore, reported WA-encapsulated liposomal formulation could be used as a therapeutic strategy to treat aggressive pancreatic Cancer [159]. WA nano vesicular system noisome formulation showed higher anticancer activity against HeLa cells in the SRB assay followed by flow cytometry and comet assays. So, this study provides an opportunity to use natural materials as cancer treatment agents [160]. Polycaprolactone implants embedded with WA was prepared for controlled systemic release to overcome the problems associated with oral bioavailability and decreasing the dose requirement. WA implant inhibits nearly 60% of lung cancer in A549 cell xenografts, but no suppression of Cancer was there when the same dose was given intraperitoneally [161]. WA formulation has proved beneficial in arthritis and Cancer, although this formulation still requires more process optimization for efficient clinical translation. This could also be beneficial for pharmaceutical and translational researchers.

## 6. Synergistic Combination of Withaferin A

The radiosensitizing combination effect of WA and hyperthermia (HT) or radiotherapy (RT) was studied (acute and fractionated) on B16F1 mouse tumors propagated in C57BL mice and Swiss albino mice [42]. Fractionated radiotherapy with WA increased the complete response (CR) of both the tumors synergistically compared to hyperthermia, further enhancing these effects. Therefore, when used with radiotherapy, WA suggested being a promising radiosensitizer. WA and myricetin (MY) combination act against the growth of pancreatic cancer. It decreased IC_50_ of WA (2.19, 2.65, 3.93 folds) compared to alone WA in PC cells (Panc-1, MiaPaca-2, BxPc-3). 1 µM of WA and 5 µM of MY, when combined, induced an increase in caspase-3 activity by 4-fold as compared to alone treatment of WA (1 µM) (2.3-fold) or MY (5 µM) (1.4-fold), which had shown less effect on the activity of caspase-3. As a whole, WA and MY’s synergism caused apoptosis in pancreatic cells [162].

The in vitro co-treatment efficacy of sorafenib (SO) and withaferin A was synergistically evaluated against papillary and anaplastic thyroid cancers. Cell viability reduced significantly from 50% (alone with each drug) to 19% (in combination). G2/M cell-cycle arrest caused by SO+WA combination in anaplastic cells and induces apoptosis (PARP cleavage and inactivation of caspase-3), down-regulating the client proteins like BRAF, Raf-1, and ERK when combined drugs were given. This research provided an approach for maintaining anticancer efficacy with a combination of SO and WA [163]. Antiproliferative and apoptotic effects were evoked by co-treatment of WA and doxorubicin in many cell lines of ovarian cancer (A2780, A2780/CP70, and CaOV3). This resulted in a reduced chemotherapeutic dose of doxorubicin, and even the doxorubicin-induced side effects were also minimized. A significant increment in the production of ROS by co-treatment resulted in damage to DNA and autophagy induced (by increased expression of autophagy marker LC3B), also through caspase-3 cleavage cell death induced. 70–80% reduction was examined in ovarian tumor cells growth xenograft of nude mice generated by synergistic WA. There was a rise in autophagy as seen by LC3B autophagy marker expression and via cleavage of caspase-3 inducing cell death. The combination regimen of WA and cisplatin at suboptimal dose generates ROS and causes cell death [41]. The actions of this combination is attributed by eradicating cells, revealing markers of cancer stem cells like CD34, CD44, Oct4, CD24, and CD117 and downregulation of Hes, Hey, and Notch genes [42].

The synergistic effect of WA and oxaliplatin combination in vitro and in vivo studies on human pancreatic cells was studied. This combination results in inhibition of proliferation and caspase-regulated apoptosis. The dysfunction of mitochondria and PI3K/AKT inhibition generates intracellular ROS through which the proapoptotic effect occurred synergistically. While in vivo methodology evaluated high synergistic antitumor activity in PC xenografts [81]. Hence, a novel pharmaceutical approach for pancreatic cancer treatment could be a cocktail of oxaliplatin and WA.

Effects of both DOXIL (liposomal preparation of doxorubicin) and WA, alone and in combination form was investigated on cell lines of ovarian cancer (A2780) and tumor growth in SCID mice. In vitro spheroids formation assay was used for studying the combinatorial effect of DOXIL and WA on the tumorigenic function of ALDH1 cells (A2780 isolated). Alone treatment of WA (dose-dependent) inhibited both ALDH1 and Notch 1 gene expression, and DOXIL (200 nM) remained ineffective. When treated in a combination of WA+DOXIL, ovarian cancer cell proliferation and ALDH1 protein expression were inhibited with a significant synergistic effect. A robust significant reduction (60% to 70%) in the growth of the tumor, as well as complete metastasis inhibition, was seen in SCID mice (with the ovarian tumor) when co-treated with DOXIL (2 mg/kg) and WA (2 mg/kg). Altogether, it was determined that WA+DOXIL (co-treatment) could be a potable factor for ovarian cancer treatment [164].

Recently, researchers investigated the WA (alone) and the combination of Paclitaxel (PAC) and WA on non-small cell lung cancer (NSCLC) cells growth, proliferation, migration, and invasion. They deceived in vitro probable effects of PAC, Cis-Pt, and WA synergistically in H1299 and A549 cells. 1:40, 1:100 and 10:1 were the combinations of ratios of PAC: WA, PAC: Cis-Pt, and Cis-Pt: WA respectively for examining synergism and further found highly synergistic, showing greater sensitivity of H1299 and A549 cells with co-treatment (PAC and WA). Colony formation, migration, the inversion was inhibited with co-treatment of PAC+WA synergistically and cause apoptosis in H1299 and A549 cells. It was demonstrated that WA targeted both drug-resistant and drug-sensitive NSCLC cells along with PAC’s synergistic effects. Hence, the anticancer role of WA alone or with PAC opposing human NSCLC cell lines was investigated, and PAC+WA combination on NSCLC provided a therapeutic strategy [165].

A study found the antitumor effect of WA with 5-fluorouracil (5-FU) by modulating endoplasmic reticulum (ER) stress feasible for cell death and inducing a significant effect antiproliferation. Autophagy and apoptosis were induced by ER stress. Co-treatment of WA and 5-FU arrested G_2_M phase cell cycle triggered due to phosphorylation of β-catenin/Wnt signaling (essential proteins). The combination treatment decreased the cell viability in colon cancer cells resulting in more robust efficacy and safe toxicity profile [166]. In the recent outbreak of SARS-CoV-2 disease, an in-silico study reported the antiviral role of within one-N combined with caffeic acid phenethyl ester and WA inhibits SARS-CoV-2 protease M^pro^ functional activity [149]. This study can lead to drug discovery for the treatment of the COVID-19 pandemic. These synergistic combinations of WA reduce toxicity associated with synthetic drugs and help design clinical chemotherapeutic strategy for human carcinoma.

## 7. Conclusions and Future Perspectives

This review has thrown light on WA pharmacokinetics, its structural modifications, and potential pharmacological activities and formulations. The pharmacokinetics studies revealed that WA’s oral absorption is rapid and can be used to design drug delivery systems targeting various diseases. The molecule is attaining global attention as it is a promising anticancer compound with many other therapeutic benefits, including AD, cardioprotective, neuroprotective, osteoporotic, and antiviral effects. In the present review, WA regulates multiple antitumor pathways, including oxidative stress, promoting apoptosis, autophagy, inhibiting cell proliferation, reducing angiogenesis progression, and metastasis progression. Identifying new proteins with significant effects on tumor progression can target future drug discovery of chemotherapeutic agents. The molecular roadmap of WA can also help us select other anticancer compounds, and their synergistic combination can boost clinical efficacy.

The combined activities of WA with radiotherapy and other chemotherapeutic drugs and the analogs of WA have shown beneficial therapeutic outcomes, thereby exploring disease-altering therapies. Moreover, an additional novel target can be designed based on the target cascade of WA; this could further have the potential for novel antitumor therapies. A single target drug develops escape pathways and has more chances to develop resistance and disease relapse. WA acts on multi targets, improves therapeutic outcomes, and overcome drug resistance.

Moreover, in-depth research on pharmacokinetics and bioavailability is needed to establish the active dose of this compound. An extensive toxicological evaluation is needed to determine the safety profile of WA. The tissue exposure of WA by pharmacodynamics biomarkers and even in vivo and in vitro studies could be performed at the same time for significant outcomes. The review evidenced that WA is a potential therapeutic approach that should be considered a potential therapeutic medicine. Validated, planned, and comprehensively designed clinical trials are imperatively needed on various cancers studies before conversion into the clinical realm.
